# Beyond Midostaurin: Role of Avapritinib in Managing Systemic Mastocytosis

**DOI:** 10.7759/cureus.60161

**Published:** 2024-05-12

**Authors:** Ngowari Pokima, Georges Khattar, Praneeth R Keesari, Salman Khan, Nnedindu Asogwa, Muhammad Niazi, Ruifang Zheng, Qun Dai

**Affiliations:** 1 Internal Medicine, Staten Island University Hospital, Staten Island, USA; 2 Hematology and Medical Oncology, Northwell Health/Staten Island University Hospital, Staten Island, USA; 3 Pathology, Staten Island University Hospital, Staten Island, USA; 4 Hematology and Medical Oncology, Northwell Health, Staten Island, USA

**Keywords:** systemic mastocytosis with an associated clonal hematological non-mast cell lineage disease (sh-ahnmd), targeted therapeutics, molecular target therapies, tyrosine kinase receptor inhibitors, systemic mastocytosis

## Abstract

We present a case of an adult male who presented with pancytopenia accompanied by symptomatic anemia, necessitating chronic transfusions. He was diagnosed with systemic mastocytosis with an associated hematologic neoplasm. Following an inadequate response to midostaurin therapy, the patient was initiated on the newly approved avapritinib. The patient showed significant improvements in all three blood cell lines; however, he developed leg edema, blepharedema, and gum bleeding on this medication. This case underscores the intricacies of managing a patient with advanced systemic mastocytosis, the emerging role of highly selective KIT inhibition in its treatment, and the practical management of adverse medication effects.

## Introduction

Mastocytosis, a rare disorder, is characterized by the clonal proliferation of mast cells in diverse organs throughout the body. These aberrant mast cells amass in the bone marrow, skin, liver, spleen, and other organs, yielding a heterogeneous clinical spectrum [1]. This ranges from restricted disease (cutaneous mastocytosis), primarily observed in the pediatric population, to a more aggressive variant with extracutaneous involvement (systemic mastocytosis) [2]. The latter is predominantly found in adult patients and, in rare instances, manifests as a malignant solid tumor marked by destructive growth, termed mast cell sarcoma [3]. 

While systemic mastocytosis is more prevalent among adults, it can manifest without skin involvement and affect individuals of any age [4]. This presents distinctive challenges for diagnosis and management. The World Health Organization (WHO) identifies five subcategories of systemic mastocytosis: indolent systemic mastocytosis, smoldering systemic mastocytosis, aggressive systemic mastocytosis, systemic mastocytosis with associated hematologic neoplasm, and mast cell leukemia [3]. The latter three represent advanced systemic mastocytosis (AdvSM), a condition with a poor prognosis with a median overall survival (OS) ranging over four years [5].

The clinical diagnosis of SM starts with a bone marrow biopsy, as this site is most frequently impacted by mastocytosis [6]. The presence of multifocal dense mast cell aggregates in histology is a major criterion for diagnosis [7]. Additional analyses involve immunotyping for CD25 and/or CD2 expression on abnormal mast cells, serum levels of tryptase [8], and molecular DNA testing for KITD816V mutation [9]. In cases presenting with blood eosinophilia, FIP1L1-PDGFRA testing is pertinent [10]. The combination of these facilitates the subcategorization of SM, guiding both prognostic assessment and therapeutic decision-making.

The management of systemic mastocytosis (SM) is notably tailored to each individual, factoring in the specific subtype, extent of organ involvement, and patient's risk profile. Treatment approaches include observation alone, symptom management (e.g., antihistamines, proton pump inhibitors), supportive measures (e.g., red blood cell transfusions, osteoporosis treatment), cytoreductive therapy (e.g., interferon alpha, cladribine) [11], and targeted tyrosine kinase inhibition (e.g., midostaurin, imatinib, avapritinib) for mast cell debulking, especially in cases of aggressive or treatment-resistant disease [12]. Other options include interferon-a in individuals who are not candidates for tyrosine kinase inhibitors (TKIs) (eg, older adults), but the response to it is generally slower [13].

In this report, we detail a case of a 67-year-old male who presented with pancytopenia accompanied by symptomatic anemia necessitating transfusions. A bone marrow biopsy confirmed systemic mastocytosis with an associated hematologic neoplasm. Following an inadequate response to midostaurin therapy, the patient was initiated on the newly approved avapritinib. Notably, this shift led to a significantly improved response to the treatment.

## Case presentation

A 67-year-old male, with a medical history of moderate intellectual disability, essential hypertension, mixed hyperlipidemia, osteoporosis, and mild cataracts bilaterally, initially presented due to pancytopenia requiring frequent transfusions to manage symptomatic macrocytic anemia. At presentation, he had a hemoglobin level of 6.4 gm/dL, platelets 120 K/μL, WBC 3.4 K/μL, and absolute neutrophil count (ANC) 1900. His initial diagnostic investigations, including assessments of iron levels, B12, folate, serum and urine protein electrophoresis (SPEP, UPEP), immunofixation studies, and free light chain assays, all yielded normal results.

Subsequently, a bone marrow biopsy was performed, revealing hypercellular bone marrow with granulocytic hyperplasia with slightly left-shifted maturation. findings indicative of myelodysplastic syndrome with multilineage dysplasia (MDS-MLD). He was initiated on erythrocyte stimulating agents (Retacrit^TM^), titrated up to 40,000 units weekly. Despite this treatment, he continued to require frequent transfusions every four weeks.

**Figure 1:**
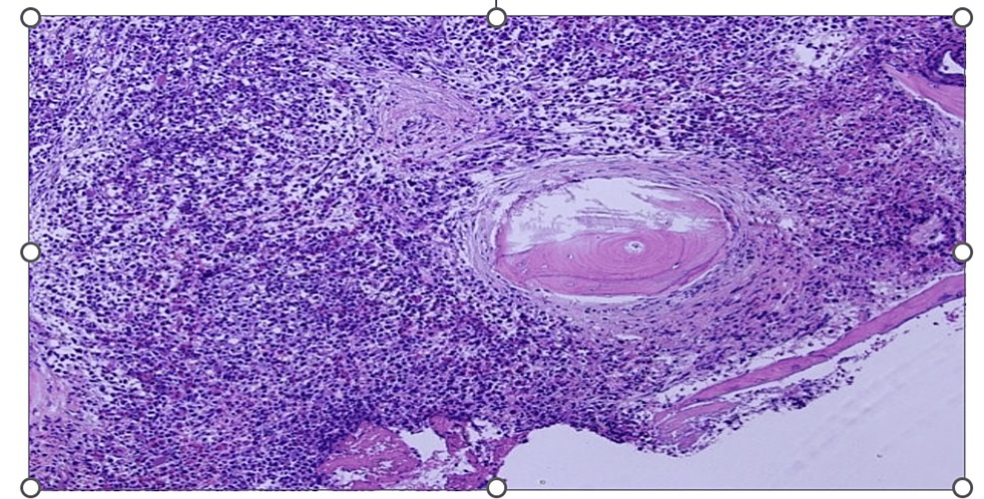
H&E sections showing clustered spindle-shaped mast cells

**Figure 2:**
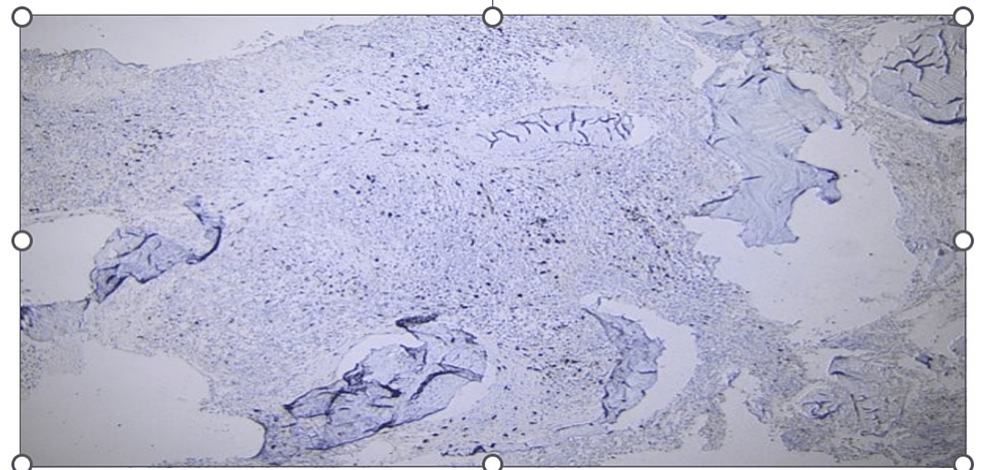
Neoplastic mast cells showing CD117 reactivity

**Figure 3:**
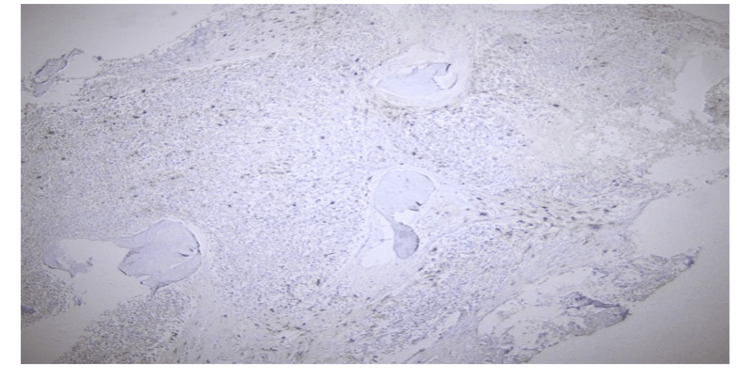
Neoplastic mast cells showing CD25 reactivity

A follow-up bone marrow biopsy conducted a year later unveiled a myeloid neoplasm with grade 3 myelofibrosis. Notably, atypical mast cell aggregates (Figure 1) were observed, which were positive for CD117 (Figure 2), CD25 (Figure 3), and CD2 (partially, weakly). Molecular analysis confirmed the presence of a KITD816V mutation while elevated serum tryptase levels reached 117 μg/L (Normal 0-11.4). Collectively, these findings were consistent with a diagnosis of systemic mastocytosis with an associated hematological neoplasm.

The patient was not a candidate for a bone marrow transplant because of his comorbid conditions and functional status (Eastern Cooperative Oncology Group (ECOG) 2). Hence, treatment with midastaurin was initiated and gradually titrated up to 100 mg q12h, which yielded limited results as he continued to necessitate transfusions every four weeks. After three months, he transitioned to avapritinib 50 mg, in line with current trends in practice, it was gradually escalated to 200 mg daily, which elicited a favorable response. He received supportive transfusions to keep his platelet count above 50 K/uL. His transfusion requirements abated for a period of five months with an improvement of hemoglobin from 6 g/dl to 12 g/dl, platelets from <20K/uL to >300k/uL, and serum tryptase level from 117 ug/l to 11 ug/l. However, the emergence of side effects, including left eye blepharedema and tearing, lower extremity edema, and gum bleeding, prompted the suspension of avapritinib. Following a reduced dose of 100 mg daily, the patient's tolerance improved, and he experienced a notable enhancement in fatigue and generalized weakness. Currently, he has sustained this reduced dosage for four months without requiring further transfusions.

## Discussion

Guidelines suggest either midostaurin or avapritinib; however, midostaurin is often the preferred first-line treatment for patients with advanced systemic mastocytosis due to its superior risk/benefit profile when compared to alternative therapies and provider experience with the drug. Functioning as a multi-kinase inhibitor, midostaurin not only targets c-KIT mutations but also other signaling pathways believed to be involved in mast cell activation [14]. The prominence of the KIT D816V driver mutation, observed in 90% to 95% of patients with systemic mastocytosis, makes it a very attractive therapeutic target [15].

Midostaurin was the first KIT inhibitor to undergo evaluation for systemic mastocytosis treatment. It yielded remarkable reductions in bone marrow mast cell burden, serum tryptase levels, and splenomegaly [5]. Additionally, it led to substantial enhancements in the quality of life, mitigating symptoms except for nausea and vomiting, which are recognized as common midostaurin-related adverse effects [14]. The compelling data resulting from these outcomes culminated in the FDA and EMA's 2017 approval of midostaurin for systemic mastocytosis treatment [16], thereby setting a significant milestone in response and survival benchmarks utilizing KIT inhibition for this condition.

Within the landscape of tyrosine kinase inhibitors, avapritinib emerges as a distinct contender, demonstrating heightened selectivity for the inhibition of D816V-mutated KIT and PDGFRA A-loop mutants when compared with midostaurin [17]. Its in vitro potency, exhibiting a 10-fold greater efficacy, underscores its potential [17]. The phase 1 EXPLORER study [18] and phase 2 PATHFINDER study [19] presently evaluate avapritinib's clinical efficacy and safety. A comprehensive evaluation combining outcomes from EXPLORER and PATHFINDER against patients receiving the best available therapy reveals that avapritinib-treated individuals exhibited significantly enhanced survival. These patients also showed the largest reductions in serum tryptase levels [20]. Furthermore, a matching-adjusted, indirect treatment comparison between the participants of midostaurin and avapritinib trials further solidified the statistically significant improvement in overall response rates, complete response rates, and survival for avapritinib over midostaurin [21].

Analysis of the phase II PATHFINDER study unveils reductions of 50% or more from baseline in serum tryptase (93%), bone marrow mast cells (88%), and KIT D816V allele frequency (60%). Impressively, 30% of patients achieved molecular remission of KIT D816V, signifying a groundbreaking advancement in the treatment of advanced systemic mastocytosis [22]. These robust outcomes ultimately led to the FDA's approval of avapritnib in 2021 for adult patients with advanced systemic mastocytosis [23]. The most encountered adverse reactions in individuals with advanced systemic mastocytosis included thrombocytopenia, anemia, neutropenia, periorbital/peripheral edema, diarrhea, nausea, fatigue/asthenia, and cognitive and other CNS side effects [19]. When initiating the treatment, the bone marrow suppressions associated with avapritinib can pose clinical challenges for physicians in treating patients with low cell counts at baseline. Patients who have cytopenias at the time of initiation of treatment are at increased risk of bone marrow suppression [19]. In the Phase 1 EXPLORER trial, the risk of grade 3 or higher thrombocytopenia was 20% in the patients without baseline cytopenia, however, this risk was increased to up to 70% in patients who had thrombocytopenia at baseline [18]. It's important to note that Avapritinib is not recommended for patients with advanced systemic mastocytosis if their platelet counts fall below 50 K/μL due to the risk of worsening cytopenias and intracranial hemorrhage [23]. In our patient, avapritinib was initiated at the dose of 50 mg and slowly escalated with supportive platelet transfusions to keep the counts above 50 K/μL.

## Conclusions

This case contributes valuably to the growing body of evidence in support of avapritinib's use in advanced systemic mastocytosis. Its remarkable capability to achieve molecular remission of c-kit mutations highlights the direct correlation between heightened c-kit selectivity and potency. Establishing standards for molecular monitoring of KIT D816V and embracing the concept of minimal residual disease (MRD) in advanced systemic mastocytosis is of great importance. Equally noteworthy is BLU-263, a KIT D816V inhibitor demonstrating equipotency with avapritinib in vitro. Its minimal central nervous system penetration suggests the potential for fewer central nervous system side effects. As we continue refining highly selective inhibition for conditions defined by driver mutations, the landscape holds promise for transformative changes in aggressive and lethal diseases.
